# Conduction system pacing in everyday clinical practice: EHRA physician survey

**DOI:** 10.1093/europace/euac201

**Published:** 2022-11-22

**Authors:** Bratislav Kircanski, Serge Boveda, Frits Prinzen, Antonio Sorgente, Ante Anic, Giulio Conte, Haran Burri

**Affiliations:** University Clinical Centre of Serbia, Pacemaker Centre, Belgrade, Serbia; Faculty of Medicine, University of Belgrade, Belgrade, Serbia; Heart Rhythm Department, Clinique Pasteur, 31076 Toulouse, France; Universitair Ziekenhuis Brussel—VUB, Heart Rhythm Management Centre, Brussels, Belgium; INSERM U970, 75908 Paris Cedex 15, France; Department of Physiology, Maastricht University, Maastricht, The Netherlands; Epicura Centre Hospitalier, Hornu, Belgium; Department for Cardiovascular Diseases, University Hospital Center Split, Split, Croatia; Cardiology Department, Fondazione Cardiocentro Ticino, Lugano, Switzerland; Cardiac Pacing Unit, Cardiology Department, University Hospital of Geneva, Rue Gabrielle Perret Gentil 4, 1205 Geneva, Switzerland

**Keywords:** Conduction system pacing, His bundle pacing, Left bundle branch area pacing, Left bundle branch pacing, Physiological pacing, Cardiac resychronization therapy, EHRA survey

## Abstract

With the increasing interest in conduction system pacing (CSP) over the last few years and the inclusion of this treatment modality in the current guidelines, our aim was to provide a snapshot of current practice across Europe. An online questionnaire was sent to physicians participating in the European Heart Rhythm Association research network as well as to national societies and over social media. Data on previous experience with CSP, current indications, preferred tools, unmet needs, and perceptions for the future are reported and discussed.

**What’s new?**

- Over half of 184 respondents had previous experience with either His bundle pacing or left bundle branch area pacing (LBBAP), indicating high adoption rates (but most of the data reflected practice in university centres).
- The main reason (75% of the respondents) for not adopting conduction system pacing (CSP) was lack of training, and only a minority (4.4%) believed that CSP was not useful.
- Conduction system pacing was the preferred pacing modality in patients with a bradycardia indication.
- Biventricular (BiV) pacing remains the preferred strategy in patients with an indication for cardiac resynchronization therapy with a left bundle branch block.
- The majority of CSP implantations are performed using lumenless leads, although most participants reported that they have also used stylet-driven leads for LBBAP.
- It is anticipated that CSP (mainly LBBAP) will predominate over conventional right ventricular and BiV pacing in the future.

## Introduction

With the advent of new tools that have increased implantation success rates,^1^ conduction system pacing (CSP) has been gaining marked interest over the last few years for providing a more physiological alternative to right ventricular pacing (RVP) and also for cardiac resychronization therapy (CRT). His bundle pacing (HBP) was introduced for the first time in the European Society of Cardiology (ESC) guidelines on supraventricular tachycardias as a Class I (Level of evidence C) recommendation for an ‘ablate and pace’ indication for treating tachycardiomyopathy.^2^ The 2021 ESC pacing and CRT guidelines expanded the use of HBP as a Class IIa (Level of evidence B) indication as rescue therapy for failed CRT implantation and defined a Class IIb (Level of evidence C) indication as an alternative to RVP in patients with left ventricular ejection fraction (LVEF) > 40% who require >20% ventricular pacing, as well as for patients with an ‘ablate and pace’ indication.^3^ No recommendations were made on left bundle branch area pacing (LBBAP) due to limited evidence at the time of writing the guidelines. A considerable number of reports have been published since then and are shaping current clinical practice. Despite not having been included in the ESC guidelines as yet, LBBAP seems to be overtaking HBP due to superior electrical thresholds, shorter procedural durations, and higher success rates.^4^ The aim of this European Heart Rhythm Association (EHRA) survey is to evaluate the current status of CSP adoption amongst device implanters.

## Methods

An online EHRA survey consisting of 22 questions was prepared by a writing group appointed by the EHRA scientific initiatives committee and distributed to members of the EHRA research network involved with device implantation. We also contacted the members of our National Societies and distributed invitations via social media platforms (Twitter, LinkedIn, and Facebook). The EHRA survey was conducted between 14 April and 22 May 2022.

### Statistical analysis

Continuous variables are presented as mean and standard deviation, while categorical variables are expressed as percentages based on available data (as it was not obligatory to fill out all the fields). Descriptive statistics were performed using Microsoft Excel (Microsoft Corporation, Redmond, WA, USA).

## Results

A total of 184 physicians filled out the online questionnaire. Each question was answered on average by 90.6 ± 8.8% of the respondents.

### Profile of the respondents and previous experience with conduction system pacing

Respondents were based in 31 countries, mostly aged 30–50 years (77.2%), with 81.2% being male, working mostly in a university hospital (69.6%) followed by in a specialized cardiology centre (12.2%). Of the respondents, the average experience with device implantation was 12.3 ± 8.5 years. Conduction system pacing had been implanted by 117 (63.6%) respondents. Both HBP and LBBAP had been implanted by 86 (46.7%) respondents, only HBP by 9 (4.9%) and only LBBAP by 22 (12.0%). Of 95 physicians who had previously implanted HBP, 32.6% had experience with > 40 implantations and 16.8% with >100 implantations. Of 108 physicians who had previously implanted LBBAP, 40.7% had experience with >40 implantations and 18.5% with >100 implantations. About half of the HBP implanters had started since ≥2 years, whereas LBBAP implantation was more recent with only a fifth having had this experience. Of the few physicians who were only implanting HBP, 7/9 (77.8%) were planning to start implanting LBBAP, whereas only 4/22 (18.2%) of physicians who were performing only LBBAP declared that they were planning to start HBP. Of 67 respondents who had never implanted CSP, 41.8% were planning on starting in the future.

Amongst respondents who gave a reason for not having yet started implanting CSP, the main motives (several possible answers) were lack of training (75.0%), lack of necessary tools at the implanting centre (33.8%), restricted lab time (22.8%) or requirement for a 12-lead electrocardiogram (ECG)/electrophysiology recording system (16.2%). Only a minority had not started yet because of lack of evidence (11.8%) or because they did not believe CSP to be useful (4.4%).

### Pacing indications among conduction system pacing implanters

**Figure 1:**
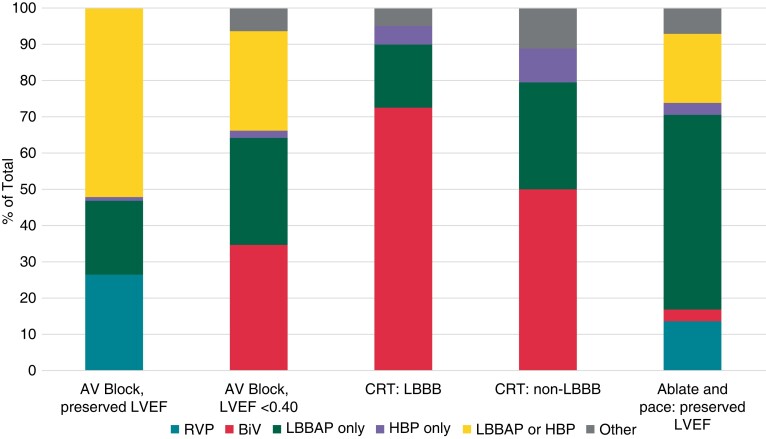
Preferred pacing modality according to indication amongst physicians who implant conduction system pacing. ‘Other’ includes His-optimized and left bundle pacing optimized CRT (HOT-CRT and LOT-CRT, respectively). AV, atrioventricular; BiV, biventricular; CRT, cardiac resynchronization therapy; HBP, is bundle pacing; LBBAP, left bundle branch area pacing; LBBB, left bundle branch block; LVEF, left ventricular ejection fraction; RVP, right ventricular pacing.

The distribution of pacing types for different indications is shown in *Figure 1*. For anti-bradycardia pacing, CSP was preferred only if ventricular pacing was frequent (>20% of the time), in 42.9% of the respondents, whereas CSP and RVP were preferred by default in 30.6 and 26.5% of the respondents respectively. On average, 44.4 ± 34.8% of all anti-bradycardia pacing was performed using CSP, but responses were very heterogenous, ranging from 1 to 100% of the cases. The majority of the respondents opted for biventricular (BiV) pacing in patients with a CRT indication and left bundle branch block (LBBB) but in only half of the patients in case of non-LBBB. On average, CSP was implanted in 33.0 ± 30.8% of the patients with a CRT indication (ranging from 0 to 90% of patients). In the case of failed coronary sinus lead implantation, 94.7% of respondents replied that they would switch to CSP.

### Technical aspects of conduction system pacing implantation

A backup lead for HBP was used in pacemaker-dependent patients by 43.7% of the respondents and by 25–30% of respondents in patients with high-grade atrioventricular block (AVB), ‘ablate and pace’ indication, high capture thresholds or sensing issues. Backup leads were never used by 14.9% of the respondents in the case of HBP, compared with 68.2% in the case of LBBAP. In the setting of LBBAP, backup leads were used in 5–15% of the patients with pacemaker dependency, high-grade AVB, ‘ablate and pace’ indication, or in case of sensing issues.

**Figure 2:**
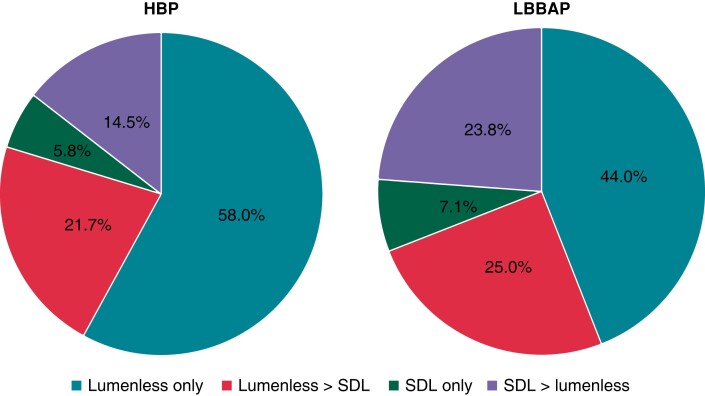
Preference of lead type according to pacing modality. HBP, His bundle pacing; LBBAP, left bundle branch area pacing; SDLs, stylet-driven leads.

Lumenless leads were predominantly used compared with stylet-driven leads (SDLs) for HBP, and to a lesser extent for LBBAP (see *Figure 2*).

### Perceptions regarding conduction system pacing

**Figure 3:**
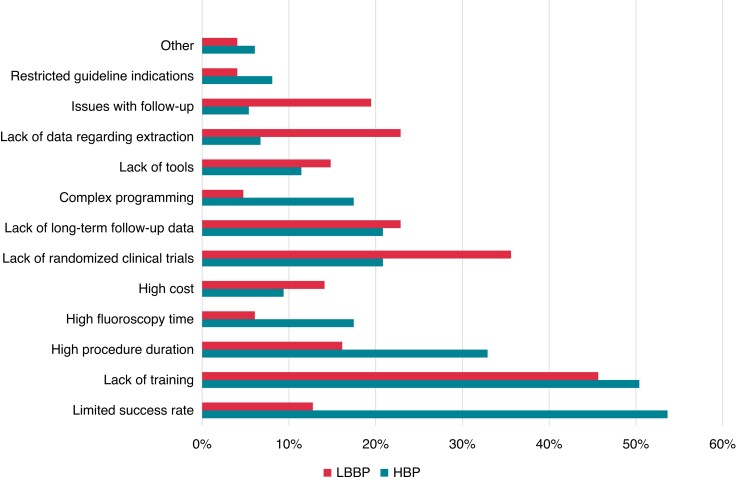
Perceived obstacles for adoption of conduction system pacing. HBP, His bundle pacing; LBBAP, left bundle branch area pacing.

**Figure 4:**
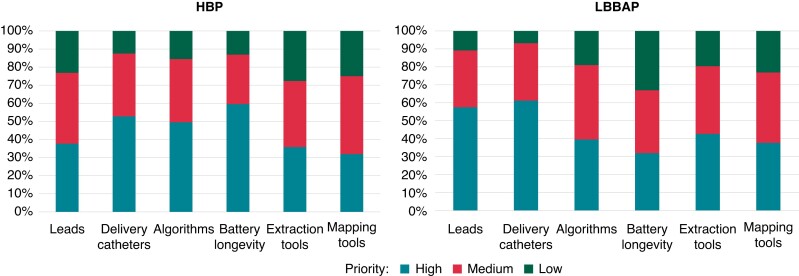
Unmet needs and priorities for innovation for facilitating adoption of HBP and LBBAP. Abbreviations as in *Figure 3*.

**Figure 5:**
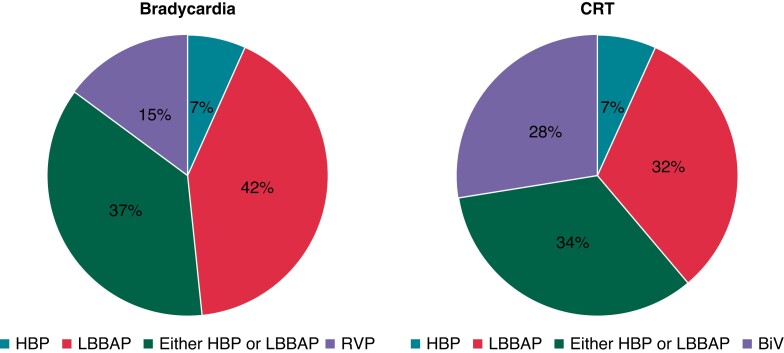
Anticipated application of different pacing modalities according to indication. Abbreviations as in *Figure 1*.

The perceived obstacles for the general adoption of CSP in the future are shown in *Figure 3*. The main hurdles for CSP follow-up (reported by about 45% of the respondents) were the lack of trained personnel, requirement for more time, and recording of a 12-lead ECG. Suggested priorities in innovation for improving CSP adoption are displayed in *Figure 4*. Overall, the majority of the respondents believe that CSP (especially LBBAP) will predominate in the future over conventional RVP or BiV pacing (see *Figure 5*).

## Discussion

Our EHRA survey indicates that over half of the respondents have performed CSP, albeit in relatively limited numbers (mostly <40 cases). The main reason for physicians to have not yet started implanting CSP was the lack of training, which was also the main obstacle mentioned as a hurdle for performing CSP follow-up and for future adoption of CSP. This underlines the need for educational and training activities in this field. The upcoming EHRA consensus document on implantation technique for CSP implantation (due in 2023) should provide a framework to standardize CSP implantation.

In the subset of physicians who had started CSP implantation for anti-bradycardia indications with requirement for frequent ventricular pacing, CSP predominated over RVP in patients with preserved LVEF and over BiV pacing in case of reduced LVEF. Overall, slightly less than half of all devices for anti-bradycardia pacing were CSP (as not all of these patients require frequent ventricular pacing). This proportion is likely to grow as the physicians gain experience and the workflow in the operating rooms gets streamlined over time for CSP implantation.

In patients with a CRT indication and LBBB, > 70% of CSP implanters continue to implant BiV pacing, which is in line with the sound evidence for this therapy from randomized controlled trials^5^ and the absence of first-line indications for CSP in CRT candidates in current guidelines.^3^ The small randomized studies^6–9^ which compared CSP with BiV pacing have shown promising results, but more data are required before CSP can become more firmly anchored in the guidelines. The question also remains if patients with non-LBBB or CRT non-responders will benefit more from CSP than from BiV pacing.^10^ Observational data from a limited number of patients are encouraging^11,12^ but require further confirmation. Some of the respondents have indicated that they combine CSP with BiV pacing. His-optimized or LBBAP-optimized CRT (termed HOT-CRT and LOT-CRT, respectively) have been shown to significantly improve electrical synchrony compared to each modality alone^13^ and exploit a synergistic effect with a fusion of activation wavefronts.

Daily clinical practice is likely to evolve before recommendations become implemented in guidelines. For example, HBP has been shown to be effective in an ‘ablate and pace’ indication^14,15^ and is currently recommended in the ESC guidelines,^2,3^ whereas this is not yet the case with LBBAP. However, due to the risk of compromising HBP thresholds by ablating near the lead tip,^16^ LBBAP is probably already being used in the overwhelming majority of patients with this indication.

Overall, LBBAP was more often used than HBP in our EHRA survey and was anticipated to predominate in the future, which is not surprising, given the reasons mentioned in the introduction. There are nevertheless advantages and limitations of both techniques.^17^ The main advantages of HBP are a narrower QRS complex^18^ and well-defined endpoints for conduction system capture.^19^ The main limitations are suboptimal electrical parameters^18,20^ and high incidence of lead revision.^21^ Left bundle branch area pacing on the other hand has excellent electrical parameters, but long-term lead survival and extractability are uncertain. The learning curve for HBP flattens out at about 40–50 cases in the hands of experienced device implanters.^22,23^ Left bundle branch area pacing success rate has been reported to be higher compared with HBP,^18^ but the definition of success is variable in different studies. The multicentre MELOS study has shown that LBBAP implantation is successful in 92.4% of bradycardia indications and in only 82.2% of heart failure indications and that the learning curve may require as many as 100 cases to flatten out.^24^ These findings support the continued use of HBP and coronary sinus pacing as alternatives in case LBBAP is not successful or does not yield favourable results.

A ventricular backup lead was used in a minority of patients with HBP, despite high-risk features such as pacemaker dependency and suboptimal electrical parameters. This is contrary to the 2021 ESC pacing and CRT guidelines,^3^ which gave a Class IIa (Level of evidence C) recommendation for a backup lead in these selected patients. The recommendation was formulated in the interest of patient safety, given the high rate of requirement for HBP lead revision.^21^ It is also surprising that backup leads were implanted with LBBAP (albeit in a minority of patients), as electrical parameters are usually excellent with this pacing modality. The practice probably reflects the initial experience during the learning curve of the operators.

Lumenless leads were preferred over SDLs, especially for HBP, as they were introduced earlier for CSP. Lumenless leads are more resistant to screw damage than SDLs when repositioning is required, especially for HBP, which targets fibrotic sites. However, SDLs are gaining popularity for LBBAP,^25^ as penetration of the interventricular septum may be facilitated by greater backup by the stylet and by stiff delivery sheaths. Most major manufacturers are currently developing tools that will facilitate implantation and probably increase SDL adoption for LBBAP.

Unmet needs and perceived priority for innovation in CSP varied slightly between HBP and LBBAP. Due to high capture thresholds that may be encountered with HBP, the requirement for increased battery longevity was stressed, followed by better delivery catheters for locating the His bundle and for automatic algorithms to facilitate programming (which can be complex).^26,27^ For LBBAP, an improved lead and delivery catheter design was the main request, most probably to facilitate challenges such as penetration of the interventricular septum.

### Study limitations

The number of participants in the EHRA survey was relatively limited. Furthermore, the majority of the respondents were based in academic centres and were experienced with over a decade of practice with device implantation. Therefore, the results of our EHRA survey are likely to be biased and may not reflect common practice.

## Conclusions

Our EHRA survey provides a snapshot on adoption of CSP and indicates that the technique has gained mainstream clinical practice in many centres. There is likely to be continued uptake of CSP (predominantly of LBBAP) as familiarity and expertise with implantation grows, new tools are developed to facilitate the procedure, and more data are published to confirm the safety and effectiveness of this treatment modality.

## Data Availability

Data are available on request to the corresponding author.

## References

[1] Zanon F , Ellenbogen KA, Dandamudi G, Sharma PS, Huang W, Lustgarten DL et al Permanent His-bundle pacing: a systematic literature review and meta-analysis. Europace 2018;20:1819–26. 29701822 10.1093/europace/euy058

[2] Brugada J , Katritsis DG, Arbelo E, Arribas F, Bax JJ, Blomstrom-Lundqvist C et al 2019 ESC guidelines for the management of patients with supraventricular tachycardia. Eur Heart J 2020;41:655–720. 31504425 10.1093/eurheartj/ehz467

[3] Glikson M , Nielsen JC, Kronborg MB, Michowitz Y, Auricchio A, Barbash IM et al 2021 ESC guidelines on cardiac pacing and cardiac resynchronization therapy. Europace 2022;24:71–164. 34455427 10.1093/europace/euab232 PMC13179788

[4] Hua W , Fan X, Li X, Niu H, Gu M, Ning X et al Comparison of left bundle branch and His bundle pacing in bradycardia patients. JACC Clin Electrophysiol 2020;6:1291–9. 33092757 10.1016/j.jacep.2020.05.008

[5] Woods B , Hawkins N, Mealing S, Sutton A, Abraham WT, Beshai JF et al Individual patient data network meta-analysis of mortality effects of implantable cardiac devices. Heart 2015;101:1800–6. 26269413 10.1136/heartjnl-2015-307634 PMC4680159

[6] Lustgarten DL , Crespo EM, Arkhipova-Jenkins I, Lobel R, Winget J, Koehler J et al His-bundle pacing versus biventricular pacing in cardiac resynchronization therapy patients: a crossover design comparison. Heart Rhythm 2015;12:1548–57. 25828601 10.1016/j.hrthm.2015.03.048

[7] Upadhyay GA , Vijayaraman P, Nayak HM, Verma N, Dandamudi G, Sharma PS et al On-treatment comparison between corrective His bundle pacing and biventricular pacing for cardiac resynchronization: a secondary analysis of the His-SYNC Pilot Trial. Heart Rhythm 2019;16:1797–807. 31096064 10.1016/j.hrthm.2019.05.009

[8] Vinther M , Risum N, Svendsen JH, Møgelvang R, Philbert BT. A randomized trial of His pacing versus biventricular pacing in symptomatic HF patients with left bundle branch block (His-alternative). JACC Clin Electrophysiol 2021;7:1422–32. 34167929 10.1016/j.jacep.2021.04.003

[9] Huang W , Wang S, Su L, Fu G, Su Y, Chen K et al His bundle pacing vs biventricular pacing following atrioventricular node ablation in patients with atrial fibrillation and reduced ejection fraction: a multicenter, randomized, crossover study. The ALTERNATIVE-AF trial. Heart Rhythm. Published online ahead of print 14 July 2022. doi:10.1016/j.hrthm.2022.07.009 35843465

[10] Herweg B , Welter-Frost A, Vijayaraman P. The evolution of cardiac resynchronization therapy and an introduction to conduction system pacing: a conceptual review. Europace 2021;23:496–510. 33247913 10.1093/europace/euaa264

[11] Vijayaraman P , Ponnusamy S, Cano Ó, Sharma PS, Naperkowski A, Subsposh FA et al Left bundle branch area pacing for cardiac resynchronization therapy: results from the international LBBAP collaborative study group. JACC Clin Electrophysiol 2021;7:135–47. 33602393 10.1016/j.jacep.2020.08.015

[12] Vijayaraman P , Herweg B, Verma A, Sharma PS, Batul SA, Ponnusamy SS et al Rescue left bundle branch area pacing in coronary venous lead failure or nonresponse to biventricular pacing: results from International LBBAP Collaborative Study Group. Heart Rhythm 2022;19:1272–80. 35504539 10.1016/j.hrthm.2022.04.024

[13] Zweerink A , Zubarev S, Bakelants E, Potyagaylo D, Stettler C, Chmelevsky M et al His-optimized cardiac resynchronization therapy with ventricular fusion pacing for electrical resynchronization in heart failure. JACC Clin Electrophysiol 2021;7:881–92. 33640346 10.1016/j.jacep.2020.11.029

[14] Su L , Cai M, Wu S, Wang S, Xu T, Vijayaraman P et al Long-term performance and risk factors analysis after permanent His-bundle pacing and atrioventricular node ablation in patients with atrial fibrillation and heart failure. Europace 2020;22:ii19–26. 33370800 10.1093/europace/euaa306

[15] Vijayaraman P , Subzposh FA, Naperkowski A. Atrioventricular node ablation and His bundle pacing. Europace 2017;19:iv10–6. 29220422 10.1093/europace/eux263

[16] Zweerink A , Bakelants E, Stettler C, Burri H. Cryoablation vs. radiofrequency ablation of the atrioventricular node in patients with His-bundle pacing. Europace 2021;23:421–30. 33241283 10.1093/europace/euaa344

[17] Burri H . Seeking the sweet spot for left bundle branch pacing. J Cardiovasc Electrophysiol 2020;31:843–5. 32043683 10.1111/jce.14378

[18] Yuan Z , Cheng L, Wu Y. Meta-analysis comparing safety and efficacy of left bundle branch area pacing versus His bundle pacing. Am J Cardiol 2022;164:64–72. 34887071 10.1016/j.amjcard.2021.10.025

[19] Burri H , Jastrzebski M, Vijayaraman P. Electrocardiographic analysis for His bundle pacing at implantation and follow-up. JACC Clin Electrophysiol 2020;6:883–900. 32703577 10.1016/j.jacep.2020.03.005

[20] Qian Z , Qiu Y, Wang Y, Jiang Z, Wu H, Hou X et al Lead performance and clinical outcomes of patients with permanent His-Purkinje system pacing: a single-centre experience. Europace 2020;22:ii45–53. 33370802 10.1093/europace/euaa295

[21] Teigeler T , Kolominsky J, Vo C, Shepard RK, Kalahasty G, Kron J et al Intermediate-term performance and safety of His-bundle pacing leads: a single-center experience. Heart Rhythm 2021;18:743–9. 33418127 10.1016/j.hrthm.2020.12.031

[22] Keene D , Arnold AD, Jastrzebski M, Burri H, Zweibel S, Crespo E et al His bundle pacing, learning curve, procedure characteristics, safety, and feasibility: insights from a large international observational study. J Cardiovasc Electrophysiol 2019;30:1984–93. 31310403 10.1111/jce.14064 PMC7038224

[23] De Leon J , Seow SC, Boey E, Soh R, Tan E, Gan HH et al Adopting permanent His bundle pacing: learning curves and medium-term outcomes. Europace 2022;24:606–13. 34849722 10.1093/europace/euab278

[24] Jastrzębski M , Kiełbasa G, Cano O, Curila K, Heckman L, De Pooter J et al Left bundle branch area pacing outcomes: the multicentre European MELOS study. Eur Heart J 2022;43:4161–73. 10.1093/eurheartj/ehac445 PMC9584750 35979843

[25] De Pooter J , Ozpak E, Calle S, Peytchev P, Heggermont W, Marchandise S et al Initial experience of left bundle branch area pacing using stylet-driven pacing leads: a multicenter study. J Cardiovasc Electrophysiol 2022;33:1540–9. 35598298 10.1111/jce.15558

[26] Burri H , Keene D, Whinnett Z, Zanon F, Vijayaraman P. Device programming for His bundle pacing. Circ Arrhythm Electrophysiol 2019;12:e006816. 30722682 10.1161/CIRCEP.118.006816 PMC6420120

[27] Starr N , Dayal N, Domenichini G, Stettler C, Burri H. Electrical parameters with His-bundle pacing: considerations for automated programming. Heart Rhythm 2019;16:1817–24. 31377421 10.1016/j.hrthm.2019.07.035

