# Corrigendum: Conduction system pacing in everyday clinical practice: EHRA physician survey

**DOI:** 10.1093/europace/euad037

**Published:** 2023-02-06

This is a corrigendum to: Bratislav Kircanski, Serge Boveda, Frits Prinzen, Antonio Sorgente, Ante Anic, Giulio Conte, Haran Burri, Conduction system pacing in everyday clinical practice: EHRA physician survey, *EP Europace*, 2022, https://doi.org/10.1093/europace/euac201

In the originally published version of this manuscript, there were errors in the chart section percentages and punctuation of the same within Figure 2. These should read:

**Figure:**
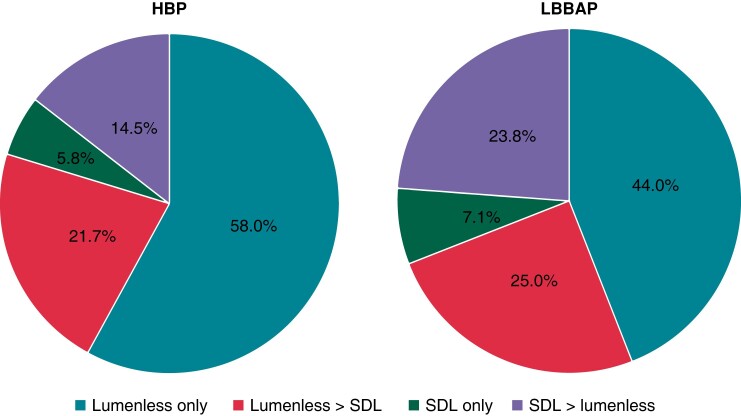


instead of:

**Figure:**
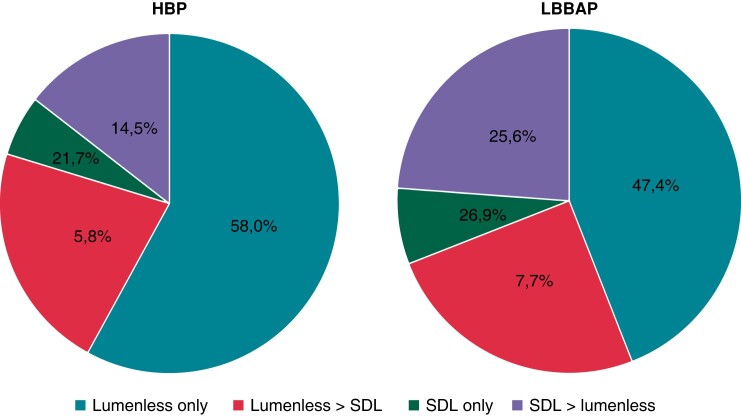


The errors have been corrected in the article.

